# Impact of antigenic evolution and original antigenic sin on SARS-CoV-2 immunity

**DOI:** 10.1172/JCI162192

**Published:** 2023-01-03

**Authors:** Muriel Aguilar-Bretones, Ron A.M. Fouchier, Marion P.G. Koopmans, Gijsbert P. van Nierop

**Affiliations:** Department of Viroscience, Erasmus Medical Center, Rotterdam, Netherlands.

## Abstract

Infections with severe acute respiratory syndrome coronavirus 2 (SARS-CoV-2) and vaccinations targeting the spike protein (S) offer protective immunity against coronavirus disease 2019 (COVID-19). This immunity may further be shaped by cross-reactivity with common cold coronaviruses. Mutations arising in S that are associated with altered intrinsic virus properties and immune escape result in the continued circulation of SARS-CoV-2 variants. Potentially, vaccine updates will be required to protect against future variants of concern, as for influenza. To offer potent protection against future variants, these second-generation vaccines may need to redirect immunity to epitopes associated with immune escape and not merely boost immunity toward conserved domains in preimmune individuals. For influenza, efficacy of repeated vaccination is hampered by original antigenic sin, an attribute of immune memory that leads to greater induction of antibodies specific to the first-encountered variant of an immunogen compared with subsequent variants. In this Review, recent findings on original antigenic sin are discussed in the context of SARS-CoV-2 evolution. Unanswered questions and future directions are highlighted, with an emphasis on the impact on disease outcome and vaccine design.

## SARS-CoV-2 immunity and memory recall upon exposure to variant viruses

The emergence of severe acute respiratory syndrome coronavirus 2 (SARS-CoV-2) resulted in the pandemic of coronavirus disease 2019 (COVID-19) (1). Symptoms are generally mild and generic for respiratory infections, including fever, cough, and myalgia (2). However, some COVID-19 patients develop more severe disease, such as acute respiratory distress syndrome, that is associated with a high mortality rate (3–5). Currently licensed vaccines offer potent protection from severe COVID-19 in naive immunocompetent individuals infected with the original SARS-CoV-2 strains (6). The vast majority of COVID-19 vaccines are based on raising immunity against a glycoprotein spike (S) that is highly similar to the surface attachment protein of the original virus strain isolated in Wuhan, China, in 2019 (1). From December 2020 onward, mass vaccination campaigns were initiated, and currently over 11 billion doses have been administered. The majority of COVID-19 vaccines induce adaptive immune responses targeting epitopes distributed over the Wuhan-Hu-1 strain S protein, with moderate differences between platforms (6, 7). Some of the S-specific antibodies can neutralize the virus particle, particularly those targeting the receptor-binding domain (RBD) or the N-terminal domain (NTD) (8, 9). These neutralizing antibodies are considered a hallmark of immune protection against SARS-CoV-2 infection and severe COVID-19 (10). CD4^+^ and CD8^+^ T cells are also thought to be essential in prevention of severe disease (11).

A footprint of adaptive immune responses to pathogens and vaccinations, including those against SARS-CoV-2 and COVID-19 vaccines, remains in the form of memory B and T cells. Re-exposure to pathogens or antigens that were encountered earlier in life will induce memory recall, where these memory immune cells tend to be boosted faster and to a greater magnitude than inexperienced naive immune cells, increasing the chance of protection from infection (12, 13). Upon exposure to variations of previously encountered pathogens or antigens, the memory B and T cell responses that target cross-reactive or shared epitopes to previous exposures will be boosted, while a response to neoepitopes is initiated. An advantage of this tendency toward cross-reactive epitopes is the natural selection of B and T cell clones that generally offer broad protection against previously encountered and upcoming related infections. However, in some cases there is a downside to this phenomenon, as was initially described for influenza A virus in 1953 (14). Here, exposure to a new influenza A virus variant of a previously encountered infection or vaccination boosted cross-reactive memory B and T cell clones that contributed little to protection, while the development of immune cell clones that target neoepitopes specific for the new variant was only modest. Thomas Francis termed this phenomenon original antigenic sin (OAS) in 1960 (15). The negative clinical impact of this phenomenon for influenza virus infection has been robustly shown in humans and various experimental animal immunization and infection studies (16, 17). OAS with a variable degree of clinical impact is found for other virus families, including dengue virus, HIV, CMV, and respiratory syncytial virus, as well as for bacterial infections (18–23).

The concept and clinical impact of OAS have been debated, and currently different interpretations of the notion of OAS still exist (23, 24). In general, what sets OAS apart from the positive effects of memory recall and cross-reactive immunity — and what inspired the use of the word “sin,” for that matter — is that OAS leads to a less potent immune response in comparison with homologous challenge or primary exposure and that this results in a competitive advantage for the variant virus.

There are other terms in the literature to describe features of a boosted memory response upon heterologous challenge that emphasize different aspects of the underlying mechanism and have a more neutral or more positive connotation than OAS (24). Whereas OAS refers to the immunological impact of the primary exposure (original antigen), the imprinting effect, or “immune imprinting,” refers to the preferential boost of cross-reactive immune cells from memory induced by prior related exposures collectively, which results in a progressively narrowed immune response toward a new strain. The extent of immune imprinting varies based on the order and the type of exposure, i.e., vaccination or mild or severe infection, and the antigenic dissimilarity between the different strains (25–29). A more neutral term to describe the boosted memory response toward strains encountered earlier in life is “antigenic seniority” (30, 31). In contrast to OAS and imprinting, antigenic seniority refers to both the positive (i.e., broad protection) and the negative contribution of past exposures to the immune response toward new exposures (imprinting). A term that focuses on the positive attributes of memory recall and its maintenance of broad immunity toward preceding strains is “back-boost,” which has been suggested to offer the prospect of preemptive vaccines for upcoming influenza strains (24, 32, 33).

OAS, imprinting effects, and antigenic seniority have been shown to modulate protection against influenza viruses in numerous independent human cohorts, including their impact to limit vaccine efficacy for prevention of infection (34–36). Despite the association of OAS and imprinting effects with a range of virus infections, birth cohorts and information from exhaustive surveillance data on co-circulation of different strains over time to determine imprinting effects of individual strains have rarely been available (34, 37). Therefore, for virus families other than influenza, imprinting effects of exposures that are assumed to have occurred earlier in life are often referred to as OAS. In the case of SARS-CoV-2, numerous variants have emerged that induce memory recall of preexisting immunity. In this Review, we focus on the mechanisms that lead to the appearance of viral variants and their relation to OAS, the molecular mechanisms behind OAS and related immune events, and the evidence of OAS in the context of SARS-CoV-2 and COVID-19.

## Evolution of SARS-CoV-2 variants

Theoretically, positive and negative side effects of memory recall can occur side by side in any new variant emerging from circulating pathogens. Nevertheless, memory recall side effects are important for specific viral pathogens from which new variants can emerge and spread globally, which leads to a large immune population. Mutations that confer intrinsic transmission advantages to the emerging variants, including mutations that allow variants to escape herd immunity by changing their antigenic properties, are selected and may allow the spread of the variant. In this context, OAS has mostly been reported for influenza viruses, which are present globally and must escape population immunity by mutating epitopes that are most vulnerable to neutralizing antibodies, resulting in the generation of immune escape variants (32, 38–40).

The ongoing evolution of SARS-CoV-2 is shown by the substantial sequence variation of virus variants that are circulating in the human population (41). Strains that rapidly became dominant in specific areas and show changes in clinical presentation, virulence, and/or transmissibility are designated as variants of concern (VOCs) by the World Health Organization, as these may impact the effectiveness of public health and social measures or available diagnostics, vaccines, or therapeutics. VOCs that emerged but are currently virtually extinct include the Alpha (B1.1.7, first detected in the United Kingdom), Beta (B1.351, first detected in South Africa), and Gamma variants (P.1, first detected in Brazil), of which the Alpha variant became dominant in Asia, Europe, and North America in the beginning of 2021. The Delta variant (B.1.617.2, first detected in India) emerged in mid-2021 and globally replaced Alpha and the majority of other VOCs before the end of 2021. At the end of 2021, the Omicron variants emerged (BA.1, first detected in Botswana and South Africa, and BA.2, first detected in South Africa) and established dominance globally. New Omicron (sub)lineages, like BA.4 and BA.5, have recently emerged and are replacing BA.1 and BA.2.

The recent VOCs are spreading in populations that are increasingly preimmune to SARS-CoV-2 as a result of vaccination or infection with preceding strains. Most slight differences between variants have had a limited impact on the effectiveness of the preexisting immunity in the population. The Omicron lineages, however, carry an unexpected high mutation load compared with the previously dominant variants Alpha and Delta, and largely escape from preexisting immunity (42, 43). The Omicron variant may have initially evolved in populations that were not covered by SARS-CoV-2 surveillance programs or in persistently infected immunocompromised individuals. In principle, variants may also originate from reintroduction to humans from an animal host, a recombination event with heterologous virus strains, or a combination of these factors, but the origins of circulating VOCs are not identified (44–46).

Besides mutations that alter intrinsic characteristics of the virus, detailed insights into the genetic changes that result in significant immune escape are imperative in order to understand which variants may become established next. This underscores the need for continuous monitoring of SARS-CoV-2 antigenic evolution and testing of vaccine efficacy for SARS-CoV-2 (41, 45).

## Molecular determinants of coronavirus antigenic evolution

Immune escape mutations are those that lead to modifications in the antigenic properties of pathogens to avoid preexisting immunity. The continuous adaptation of viruses in their antigenic properties is known as antigenic evolution. To date, the antigenic evolution of SARS-CoV-2 is primarily described for S, since this is the main target of COVID-19 vaccines and an immunodominant target during SARS-CoV-2 infection.

**Figure 1:**
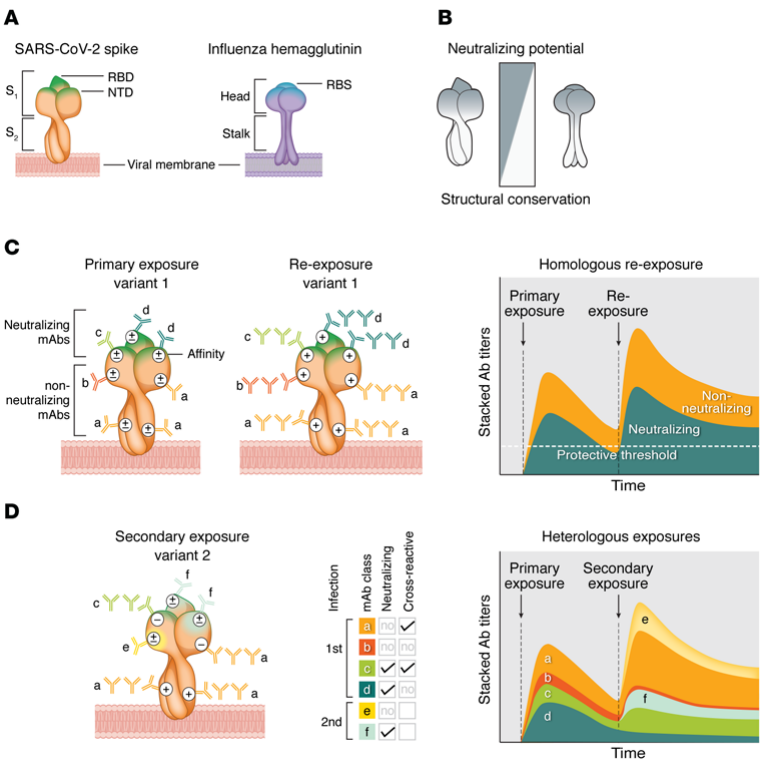
Antigenic changes of spike and hemagglutinin result in boost of non-neutralizing antibodies. (**A**) The SARS-CoV-2 spike trimer (S) consists of the S_2_ and S_1_ subdomains. S_1_ contains the N-terminal domain (NTD) and receptor-binding domain (RBD). Influenza hemagglutinin (HA) consists of a stalk domain and a head domain, which contains the receptor-binding site (RBS). (**B**) The S_2_ and HA stalk are highly conserved between virus variants, while the S_1_ and HA head are more variable. Antibodies that target S_1_ or HA head domains, especially NTD, RBD, and RBS, have the highest neutralization potential, while antibodies targeting S_2_ or HA stalk have lower neutralization potential. (**C**) Neutralizing (green color family; c and d) and non-neutralizing antibodies (orange color family; a and b) are induced after primary exposure. After exposure with the same virus variant (homologous re-exposure), both antibody classes are boosted from immune memory and undergo similar affinity maturation, from moderate (±) to high affinity (+). The kinetics of these responses is shown as a stacked plot in the right panel. (**D**) Infection with a heterologous virus strain that carries immune escape mutations in the S_1_ domain (light green and yellow) boosts cross-reactive antibodies. Those targeting shared epitopes will mature into high-affinity antibodies (+), and those that target mutated epitopes will bind with low affinity (–). Because of higher similarity in epitopes of non-neutralizing antibodies (orange; a), they are preferentially boosted over neutralizing antibodies (green; c). Neoepitopes are targeted with moderate affinity, and these antibodies represent a minor fraction of the total response (±; e and f). The kinetics of the response is shown in the right panel. Owing to original antigenic sin, the breadth and magnitude of the neoepitope-specific response (non-neutralizing: yellow, e; neutralizing: light green, f) are lower than those of the initial response.

Although they are antigenically distinct, there are striking parallels between the antigenic evolution of S of human coronaviruses (HCoVs) and hemagglutinin (HA) of influenza viruses. Both S and HA are required for binding and fusion with the target cell and are dominant targets for neutralizing antibodies (47–50). S protrudes from the viral membrane in trimeric form and consists of the membrane-proximal domain S_2_, containing the fusion apparatus, and the outer domain S_1_, containing the RBD. Homology between S of SARS-CoV-2 variants is higher in S_2_ compared with S_1_, which is most variable in the RBD and the NTD (40). Influenza HA is also a trimeric surface glycoprotein and has a stalk domain that is highly conserved compared with the more variable head domain that contains the receptor-binding site (HA-RBS) (Figure 1A). Both S and HA are highly immunogenic, and antibody epitopes are defined covering their entire structure. A minority of antibodies bind S-RBD or HA-RBS, which contain the targets for most potent neutralizing antibodies. However, some neutralizing antibodies have also been described targeting the conserved S_2_ or HA stalk (51–53) (Figure 1B). The similarities in antigenic evolution between influenza virus HA and coronavirus S suggest that for both virus families, in contrast to reinfection with the same strain, an infection with a heterologous strain or antigenic drift variant may preferentially boost non-neutralizing antibody clones, which makes the infected individual’s immune system at risk for OAS (Figure 1, C and D).

While the significance of individual SARS-CoV-2 VOC mutations in the antigenic evolution of S largely remains to be determined (40), key amino acid substitutions driving immune escape have been identified for one related virus, the alphacoronavirus HCoV-229E, and for influenza viruses. Signature mutations defined for drift variants of these viruses are generally located in or near the S-RBD and HA-RBS, respectively (39, 54, 55). Neutralizing antibodies against these sites are most effective by directly blocking critical receptor interactions. However, specific mutations can apparently occur around these sites without detrimental effects on virus fitness (39, 54, 56). The evident accumulation of mutations in the RBD and NTD in VOCs suggests that for SARS-CoV-2, antigenic evolution of S may be similarly driven by escape from antibody neutralization (40, 54). However, additional VOC signature mutations are located elsewhere in S in the viral genome and may reflect adaptations beyond immune escape, such as improved viral fitness or altered affinity for the ACE2 receptor (57). Some may reflect compensatory mutations that are indirectly linked to immune escape (40). Additional mutations at sites targeted by antibodies with Fc-mediated functions, like complement-dependent cytotoxicity, have also been characterized. However, these mutations are generally not selected at a population level and are therefore considered less influential on antigenic evolution (58, 59). A fraction of Wuhan-Hu-1–specific T cells has reduced reactivity toward Omicron, due to mutated epitopes in S_1_ (60). However, these differences are not observed when T cell reactivity targeting the entire S is analyzed (61–63).

Together, this evidence supports that the antigenic evolution of SARS-CoV-2 S is strongly driven by antibody neutralization and resembles that of HCoV-229E S and influenza HA. However, a key difference is that SARS-CoV-2 and HCoV-229E bind the protein receptors ACE2 and human aminopeptidase N, respectively, while influenza virus binds glycan receptors (64, 65). This may differentially impact the evolutionary freedom and speed of both virus families (54, 66, 67). The types of changes that may be incorporated in SARS-CoV-2 variants near the RBD and NTD have been studied, but the rate at which they may occur and be able to spread in the human population remains unknown (66).

## Antigenic cartography to visualize viral antigenic evolution of SARS-CoV-2

**Figure 2:**
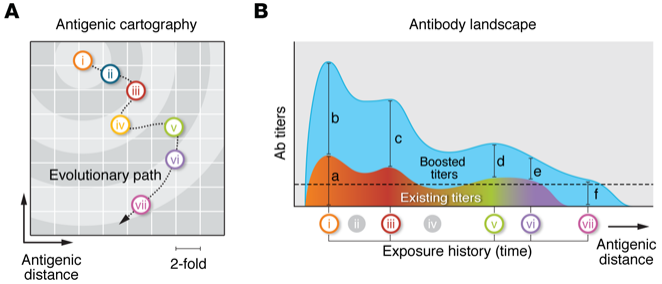
Antigenic cartography of a model virus and antibody landscape induced by heterologous exposures. (**A**) Antigenic cartography of a model virus shows the evolutionary path driven by positive selection of immune escape mutations that resulted in viral variants i–vii. The antigenic distance between variants is calculated using the virus cross-neutralization potential of sera. Each square represents a 2-fold change in virus neutralization titer. (**B**) The exposure history by infection or vaccination (variants i, iii, v, and vi) defines the existing antibody landscape (rainbow colors). OAS refers to the immunodominant response (a) of the primary exposure (i) and the negative impact this has on the breadth and magnitude of each successive exposure (i.e., iii, v, and vi). Antibody imprinting refers to the progressively narrowed immune response to each successive antigenically related exposure (i–iii, iii–v, and v–vi) due to the preferential boost of cross-reactive clones. Antigenic seniority refers to the dominant impact of older exposures on the development of a new response (i > iii > v > vi). Upon exposure to variant vii, the original antigen will be preferentially boosted (b). The boost of each successive response will be progressively less strong according to antigenic seniority (b > c > d > e). The positive effect of infection with variant vii on protection against previous strains (i–vi) is termed back-boost. The horizontal line depicts the threshold of immune protection.

Because of the dominant effect of neutralizing antibodies on the antigenic evolution of coronaviruses and influenza viruses, the level of serum antibody cross-neutralization has been used to define antigenic drift variants (68–70). Such cross-neutralization titers can be used to calculate the “antigenic distance” between different virus variants and antisera raised against them, which are visualized in “antigenic maps” (70) (Figure 2A). When supported with viral genome sequence data, antigenic maps have yielded important new insights into the molecular determinants of antigenic change. Although antigenic data are preferably generated with human sera, the use of sera from controlled animal infections has offered a powerful tool to monitor the evolutionary changes of several viruses in antigenic maps. Moreover, various antibody assays can be employed for this cartography. For various influenza viruses, detailed and robust antigenic maps were generated using serum hemagglutinin inhibition (HI) titers as a surrogate for virus neutralization assays (70). For coronaviruses, plaque reduction neutralization tests have been used to determine the antigenic relatedness of different variants (68, 69).

Influenza A/H3N2 and HCoV-229E were shown to evolve along paths where new variants repeatedly replaced the predecessors (54, 70). The influenza B virus lineage has split into two distinct evolutionary branches originally based on B/Victoria-like and B/Yamagata-like strains (71). For the influenza viruses, new variants emerge approximately every 3 to 5 years (38, 70).

Currently, there are multiple SARS-CoV-2 variants that co-circulate, and it is unclear which will go extinct, continue to coexist, or evolve into new variants. Antigenic maps of SARS-CoV-2 VOCs showed that there is substantial antigenic distance between VOCs (68, 69, 72). Variants that preceded the current VOCs (Delta, Omicron BA.1 and BA.2) are antigenically more similar to the original Wuhan-Hu-1 strain, with the exception of the Zeta variant (69). Omicron variants are currently the most distant lineage from Wuhan-Hu-1 (55, 69). Strikingly, the Delta variants are antigenically positioned opposite to the Omicron variants from the original strain, which may complicate vaccine development. For influenza virus antigenic maps, specific drops in HI titers on the antibody landscape are correlated with vaccine failure or inefficacy (73). Finding such correlation for SARS-CoV-2 VOCs would be of importance in the development of second-generation SARS-CoV-2 vaccines. Notably, as we do not have a full understanding of what confers immune protection and what are the evolutionary restraints of S, there may be vulnerable antigenic sites that can be targeted by universal vaccines (74, 75).

Cross-neutralization potential — which can be inferred from antigenic cartography — has a strong impact on an individual’s level of immune protection and development of an immune response upon successive infections and vaccinations with antigenically related virus strains. The signature antibody repertoire induced by an individual’s infection and vaccination history has been termed the antibody landscape (32) (Figure 2B). Antibody landscapes are influenced by the order and type of exposures, e.g., vaccination or infection, and severity of disease, and are subject to change upon vaccination or infection with a new antigenically related strain (32, 76). Thereby, the antibody landscape of an individual at the time of exposure has a profound impact on the level of immune protection and the type of immune response evoked upon infection with a new variant virus. This includes potential effects on OAS, imprinting, antigenic seniority, and back-boost (Figure 2B).

## Immunological mechanisms of original antigenic sin

Upon secondary infection with a heterologous strain, the level of antigenic relatedness in the amino acid sequence, antigen confirmation, and glycan composition defines the magnitude, functionality, and breadth of the recalled memory response and, potentially, OAS. For both T cells and B cells, OAS is affected by (a) the kinetics, i.e., the speed and magnitude of the memory versus naive B and T cell responses; (b) the affinity and functionality of the immune response; and (c) the immunological breadth of the response at the time of exposure.

For CD8^+^ T cells, memory recall and a delayed de novo response may occur because human memory CD8^+^ T cells have higher immunological synapse propensity than naive CD8^+^ populations (13). This gives memory CD8^+^ T cells a competitive advantage over naive CD8^+^ T cells. Notably, this difference between memory and naive phenotypes is observed neither in human CD4^+^ T cells nor in murine T cells. However, in experimental HIV and CMV immunization and infection studies in mice, activation of CD8^+^ T cells with a variant epitope impairs their function, resulting in reduced activation, proliferation, and cytokine production and delayed viral clearance (19, 77). In humans, repeated dengue virus infections with a heterologous strain may lead to activation of CD8^+^ T cells with lower affinity for the current versus prior infecting strains and to a clonally less diverse response (78, 79). This effect is correlated with specific HLA class I alleles, which may in part explain a genetic predisposition for severe dengue disease and OAS (79).

In the case of CD4^+^ T cells, no evidence supports direct functional implications of imprinting. Nevertheless, the magnitude of newly induced CD4^+^ T cell responses declines after repeated heterologous challenge (80–82). This might be explained by a selective recruitment of memory CD4^+^ T cells, which results in reduced immunological CD4^+^ T cell breadth (81, 82). This reduced CD4^+^ T cell repertoire may subsequently fuel the extent of antibody imprinting by limiting T helper functions to B cells in germinal centers (GCs). Currently there is limited evidence of CD4^+^ or CD8^+^ T cell epitope escape for SARS-CoV-2 variants (61–63). However, it remains to be determined whether CD4^+^ and CD8^+^ T cell immunity specific to HCoVs and highly pathogenic CoVs, such as the betacoronaviruses SARS-CoV and Middle East respiratory syndrome coronavirus (MERS-CoV), induces imprinting effects on SARS-CoV-2 immunity or vice versa.

Memory B cells also possess an intrinsic proliferative advantage over their naive B cell counterparts (12). As a result, proliferating plasmablasts generated upon a heterologous flavivirus immunization preferentially target preceding flavivirus infections in animal models and in natural flavivirus infections in humans (83–85). Heterosubtypic infections may also boost antibodies that inefficiently bind the boosting antigen through multiple low-affinity interactions with the surface B cell receptor that is expressed at high density on memory B cells. Consequently, the soluble antibody that is produced may not significantly bind the target antigen (86, 87). Alternatively, preexisting antibodies that bind conserved epitopes mask neoepitopes and thereby prevent the formation of a de novo B cell response (88, 89).

In contrast to T cells, memory B cells can mold their specificity by secondary GC reactions for further affinity maturation with the help of follicular helper T cells (Tfh cells). When the antigenic distance between the priming and the boosting variant is limited, memory B cells can be affinity trained to also target the new strain (29, 90). However, Tfh cell numbers are markedly decreased in severe COVID-19 patients (91). Consequentially, the GC reaction is impaired, which prevents affinity maturation of both preexisting and new B cell clones toward SARS-CoV-2. Instead, severe COVID-19 patients mount an elevated memory B cell response and strong extrafollicular B cell response that consists of clones with close to germline B cell receptors, indicating little affinity maturation (92).

## Original antigenic sin in SARS-CoV-2 immunity and COVID-19 vaccination

**Figure 3:**
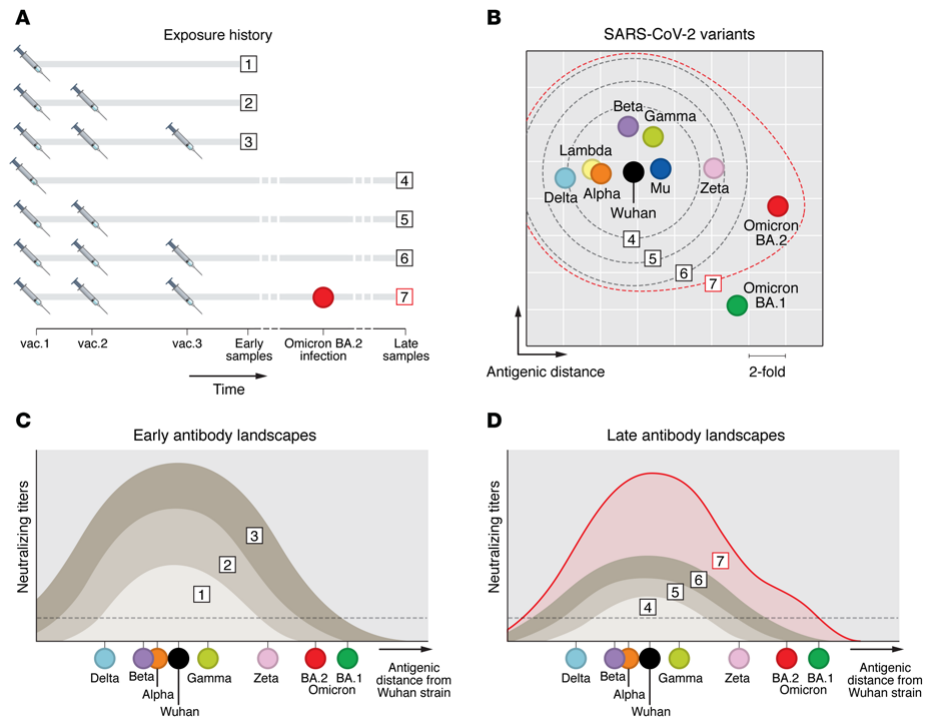
Model to correlate antigenic distance between SARS-CoV-2 variants with vaccine failure. (**A**) An individual’s signature history of exposure to SARS-CoV-2 infection and vaccination determines the level of protection from infection. Model sera are collected early (samples 1–3) and late (samples 4–6) after full vaccination and after breakthrough infection with Omicron BA.2 (sample 7, red circle). (**B**) Antigenic cartography of SARS-CoV-2 shows the antigenic distance between variants (map adapted with permission from ref. 69). (**C**) The antibody landscape shortly after first, second, and third vaccination defines the magnitude and breadth of immune protection against SARS-CoV-2 variants (samples 1–3 from **A**) based on the antigenic distance from the vaccine strain that is inferred from the antigenic map. (**D**) At a later time point, waning antibody titers (samples 4–6 from **A**) may result in poor protection from antigenically distant strains. Breakthrough infection with Omicron BA.2 (sample 7) boosts the memory vaccine response and initiates a new type-specific response (red). The dashed lines in **B** represent the immune protection threshold after vaccination or breakthrough infection based on the later antibody landscapes from samples 4–7 that are modeled in **C** and **D.**

The effect of original SARS-CoV-2 strain immunity induced by vaccination or infection on the protection against new VOCs is under debate. Although the earlier VOCs (Beta, Gamma, and Delta) carried signature mutations associated with immune escape from neutralizing antibodies, convalescent and post-vaccination sera with high titers still neutralized the variants (68, 93). However, although many vaccines offer a broader S-specific response toward variants than infection with original SARS-CoV-2, these vaccines induce limited cross-neutralization against the most antigenically distant VOCs, such as the Omicron variants (55, 62). Individuals who received three vaccine doses have 10- to 20-fold higher neutralizing titers against distant Omicron variants than individuals who received two doses (55, 94). Moreover, a third mRNA vaccine boost induced higher breadth and neutralization potency of the B cell memory response, with increased numbers of clones targeting highly conserved epitopes of RBD in comparison with two-dose recipients (95). Nevertheless, many vaccinated individuals are still susceptible to infection and to developing COVID-19 after Omicron infection (96) (Figure 3A).

The longevity of protection against VOCs by solely homologous boosting may be limited because of the potentially continuous antigenic evolution of SARS-CoV-2 and waning immunity. As a result, antibody titers sufficient to cross-neutralize future VOCs may only be reached for a short period after vaccination or boost with original S antigens. Another concern is that homologous boosting of original S-specific responses by repeated vaccination or Wuhan-Hu-1 infection may induce imprinting of the original strain and therefore result in an OAS type of response when challenged with VOCs (Figure 3, B and C). Indeed, vaccinated individuals infected with the Alpha or Delta variant have a relatively decreased response to variant-specific epitopes compared with unvaccinated individuals, which is indicative of OAS (97). However, hybrid immunity induced by combination of vaccination and infection increases the overall titers with capacity to bind to and neutralize VOCs, including Omicron, as compared with two-dose and three-dose immunizations (98–103). Thus, potentially, breakthrough infections, which are generally mild, may offer sufficient protection against current and upcoming variants (102–104). However, relying on this protection will come at the risk of long COVID-19 symptoms and will pose risks for vulnerable groups like elderly or immunocompromised people and people with underlying disease. In addition, more traits of immune imprinting have recently been identified in hybrid-immune individuals who were infected with Wuhan-1 strain before vaccination, in whom enhancement of VOC cross-reactive antibody titers and T cells by Omicron infection was nullified, a phenomenon termed hybrid immune damping (60). These studies confirm that combinations of exposures to VOCs may not always result in the exact same antibody landscapes and protection potential.

Heterologous vaccination strategies using VOC-specific vaccines are tested in small cohort studies and animal models for their efficacy and safety. Individuals who were vaccinated twice with mRNA-1273 (original S) were boosted with homologous vaccines or the Beta variant mRNA vaccine mRNA-1273.351 or a mixture thereof. Although preliminary results suggest that the vaccines were safe and the overall neutralizing titers were increased for all study participants, neutralization of the Beta variant was not significantly better upon administration of the variant boost (105). Also, in a macaque model, vaccination with Ad26.COV2.S (original S) followed by a heterologous boost with Ad26.COV2.S.351 (Beta variant) did not result in significantly elevated neutralization titers for Beta and Omicron variants, compared with a homologous boost. Moreover, non-neutralizing S-binding titers were preferentially boosted over neutralizing and RBD-binding antibody titers (106). Potentially, a second heterologous boost would improve titers toward the variants, as the titers after primary vaccination are generally modest. Nevertheless, these observations strongly support imprinting of non-neutralizing antibodies induced by the original vaccine and indicate OAS.

Although homologous and heterologous boosts with SARS-CoV-2 spike vaccine and hybrid immunity resulted in increased neutralization titers against VOCs, it is important to monitor how well immunity can be shaped to target neoepitopes in VOCs. Owing to extensive GC reactions that induce somatic hypermutation and memory B cell turnover, broadly reactive neutralizing antibodies are selected and maintained in the repertoire that potentially protect against VOCs (75). Notably, it has already been reported that pre-boost serum antibody titers against original S inversely correlate with post-boost VOC antibody reactivity (107). This indicates that high antibody titers against the original strain result in reduced immunogenicity of the variant protein, potentially by epitope masking or antigen trapping (32, 108). This will increase imprinting effects due to a reduced immune response against emerging VOCs. Therefore, effective generation of robust VOC-neutralizing antibodies with the heterologous vaccines may be achieved only when existing original S-specific serum antibody titers are waning. Most of the research assessing VOC-neutralization potential of individuals with hybrid immunity, such as those infected with Omicron variant after vaccination, is performed early after infection when GC reactions, clonal expansion of plasmablasts, and antibody maturation are still ongoing. These results will unlikely reflect the serum or memory B cell response at immune convalescence, which was shown to take up to at least 6 months and potentially even longer (74, 75, 109). Moreover, despite observations that homologous or heterologous COVID-19 vaccine prime-boost regimes generally induce prolonged GC reactions and higher magnitude of neutralizing titers toward the same or heterologous strains, the breadth of the antibody response is limited as compared with natural infection, which will also impact imprinting effects and future options for new vaccines (110). Studies with longer follow-up are required to determine long-term effects of hybrid immunity on protection against VOCs.

## Original antigenic sin in immunity to other human coronaviruses

Preexisting serum antibodies and B and T cells that can recognize SARS-CoV-2 have been detected in naive, unvaccinated individuals. These responses are most likely shaped by prior infections with the widespread seasonal human coronaviruses (HCoVs) that cause common cold symptoms, including the alphacoronaviruses HCoV-229E and HCoV-NL63 and the betacoronaviruses HCoV-OC43 and HCoV-HKU1 (111, 112). A very small percentage of the SARS-CoV-2–naive population is preimmune to the highly pathogenic betacoronaviruses (hpCoVs) SARS-CoV, which caused an outbreak around 2003, and MERS-CoV, which was discovered in 2012 and still causes zoonotic infections in the Middle East and North Africa (113, 114). These HCoVs and hpCoVs share various degrees of sequence and structural homology with SARS-CoV-2 (115) that largely correlate with the level of cross-reactivity in B and T cell immunity.

Despite the high seroprevalence of seasonal HCoV, there is high susceptibility to SARS-CoV-2 infection in unvaccinated individuals, indicating that seasonal HCoV immunity offers limited cross-protection (116). Nevertheless, the development of an immune response against SARS-CoV-2 is influenced by preexisting HCoV immunity and, in a very small percentage of the population, hpCoV immunity. Besides the development of a new type-specific response after SARS-CoV-2 infection, preexisting HCoV antibodies are boosted (87, 93, 117, 118). In particular, HCoV-OC43 S_2_–specific IgG titers are boosted in serum, but these antibodies are associated neither with protection against SARS-CoV-2 or neutralization, nor with HCoV-OC43 neutralization (87, 93, 117). Activation and proliferation of preexisting HCoV-OC43 S_2_–specific B cells result in the production of antibodies with limited detectable cross-reactivity to SARS-CoV-2 S trimer (87). This HCoV-specific boost effect is also seen upon administration of COVID-19 mRNA vaccines, although not as prominently as for natural infection (119). This boost is most prominent in patients with severe COVID-19 (87, 93, 117, 118). Moreover, studies in severe and fatal COVID-19 cases showed indications of immune imprinting and impairment of the de novo SARS-CoV-2 type-specific response (93, 117). These negative associations of seasonal HCoV-specific immunity with COVID-19 severity show features of OAS.

Therefore, the magnitude and breadth of SARS-CoV-2–specific immune responses after a primary infection are strongly influenced by the prior HCoV and hpCoV infections — the course and extent of which vary per person — and COVID-19 vaccination status, which is relatively homogeneous as current coronavirus vaccines are based on the same S. How the antigenic distance between HCoVs and hpCoVs relates to SARS-CoV-2 VOCs is currently unknown, as antigenic maps of these viruses have not been generated. Follow-up studies on potential imprinting effects and the clinical impact of SARS-CoV-2 vaccination on HCoV immunity in children may be of interest.

## Concluding remarks and future directions for SARS-CoV-2 vaccination

Current vaccines are less potent against recently circulating SARS-CoV-2 variants that emerged in partially immune populations and that are antigenically distinct from the initial SARS-CoV-2 Wuhan-Hu-1 strain (61, 120). If vaccine potency worsens, the need may arise for alternative vaccination strategies, such as periodic vaccine updates, similar to the strategy of vaccine updates for influenza, or the development of more broadly effective second-generation vaccines. With the potential continuous emergence of antigenic drift variants of SARS-CoV-2 that escape from immunity elicited by vaccination and infection, methods to overcome or limit potential negative effects of OAS should be considered in new vaccination strategies.

Research on how to overcome OAS has been performed in the context of influenza. First, the combined administration of updated heterologous vaccination with particular dendritic cell–activating adjuvants, such as *Bordetella pertussis* toxin or squalene-based oil-in-water nanoemulsion, was shown to break OAS effects in animal models (121). Current mRNA and viral vector COVID-19 vaccine platforms remain to be compared in their potential to activate dendritic cells and their effect on individuals with imprinted immunity.

Second, long-interval prime-boost regimes with 6 to 12 months between immunizations increased vaccine efficacy in vaccine studies for H5 influenza strains and may decrease the impact of OAS (90, 108). Antigen trapping and restrictions in GC capacity in early boost immunization may hamper the antibody response to new antigens (122). Future cohort studies for COVID-19 vaccines should evaluate the differences between time-spaced administrations of homologous or heterologous COVID-19 vaccines to improve the magnitude, breadth, and functionality of the adaptive immune response (97).

Third, simultaneous immunization with multiple variant antigens may prevent OAS effects in T cells and B cells, avoiding negative interference between antigens (77). This supports the importance of continued tracking of SARS-CoV-2 evolution patterns, e.g., using antigenic cartography, to design vaccines that include all circulating variants as immunogens with adjuvants (121). Simultaneous immunization of circulating strains has been proposed as a solution for universal protection against influenza virus when applied in childhood (123). SARS-CoV-2–naive populations or individuals with highly waned immunity might also benefit from simultaneous immunization with antigens of SARS-CoV-2 circulating VOCs. Initial studies on bivalent COVID-19 mRNA vaccines (combinations of Wuhan-1 and Omicron BA.1/BA.4/BA.5 S) have collected promising data such as increased breadth of neutralizing antibodies and decreased lung inflammation upon challenge in comparison with the administration of homologous mRNA-1273 boosters (124).

Fourth, removal of conserved domains from an immunogen may help overcome OAS in imprinted individuals. In an original full-length S preimmune macaque model, animals were immunized with an adjuvanted Beta variant RBD or original RBD. Here, in the absence of the S_1_ or S_2_, the Beta-neutralizing response was effectively boosted using Beta-RBD. After adoptive transfer in mice, Beta-RBD–boosted immune sera provided broader protection against severe disease upon wild-type or Beta variant challenge infection (125).

Fifth, in contrast to a variant-targeted approach, broadly reactive or even universal vaccines are also being investigated. Broadly cross-protective antibodies and B cell receptors specific for HA head domain and HA stalk have been identified in humans and are being considered as targets for influenza virus universal vaccines (126, 127). Comparable targets of broadly reactive SARS-CoV-2–neutralizing antibodies within RBD, NTD, and S_2_ have been identified (74, 75). Potentially, future COVID-19 vaccine design could be based on masking or removing variable SARS-CoV-2 S_1_ domains and relying on these more conserved sites, offering the potential positive effects of back-boost. Alternatively, vaccines that induce broad T cell immunity with the addition of peptides that represent conserved CD4^+^ and CD8^+^ T cell epitopes from S_2_ and other viral proteins may offer broad protection from SARS-CoV-2 VOCs (128). However, potential interference of SARS-CoV-2 vaccines with HCoV immunity would have to be carefully investigated before their administration. Similarly, potential impacts of SARS-CoV-2 immunity on the circulation and evolution of other HCoVs should be monitored. Studies to elucidate which of the previous strategies is the most effective in the context of SARS-CoV-2 immunity remain to be performed.

The availability of extensive biobanks and SARS-CoV-2 surveillance data may allow us to characterize, and potentially predict, the balance between cross-protective and OAS-type responses at a population level. Easily adaptable vaccine platforms, like current mRNA vaccines, offer a unique opportunity to investigate (subsequent) combinations of immunizations to optimize minimal imprinting effects and maximal effectiveness against SARS-CoV-2 variants and other virus families with complex antigenic properties. Investigation of heterologous priming and boosting exposures with the current knowledge regarding COVID-19 vaccination could turn OAS into positive back-boost effects.

## Version 1 01/03/2023

Electronic publication
